# Comparative efficacy and safety of biosimilar infliximab and other biological treatments in ankylosing spondylitis: systematic literature review and meta-analysis

**DOI:** 10.1007/s10198-014-0593-5

**Published:** 2014-05-16

**Authors:** Petra Baji, Márta Péntek, Sándor Szántó, Pál Géher, László Gulácsi, Orsolya Balogh, Valentin Brodszky

**Affiliations:** 1 Department of Health Economics, Corvinus University of Budapest, Fővám tér 8, 1093 Budapest, Hungary; 2 Department of Rheumatology, Flór Ferenc County Hospital, Semmelweis tér 1, 2143 Kistarcsa, Hungary; 3 Division of Rheumatology, Institute of Medicine, University of Debrecen Medical and Health Sciences Center, Nagyerdei krt 98, 4032 Debrecen, Hungary; 4 No. I. Department of Rheumatology, Budai Irgalmasrendi Hospital, Frankel Leó út 54, 1023 Budapest, Hungary

**Keywords:** Ankylosing spondylitis, Biological drug, Biosimilar pharmaceuticals, Meta-analysis, Efficacy, Safety, I10, I19

## Abstract

**Objectives:**

To compare the efficacy and safety of infliximab-biosimilar with other biological drugs for the treatment of active ankylosing spondylitis (AS).

**Methods:**

Systematic literature review for randomized controlled trials (RCTs) with adalimumab, etanercept, golimumab, infliximab and infliximab-biosimilar in AS was performed and indirect meta-analysis (Bayesian mixed treatment comparison) was carried out. The proportion of patients reaching 20 % improvement by the assessment of Spondyloarthritis International Society response criteria (ASAS20) at weeks 12 and 24 was used as efficacy endpoints, and the occurrence of serious adverse events at week 24 was applied to compare the safety of the biologicals.

**Results:**

Altogether, 13 RCTs, identified by the systematic literature search, were included in the analysis. Results on the ASAS20 efficacy endpoint were reported for week 12 in 12 RCTs involving 2,395 patients, and for week 24 in 5 RCTs comprising 1,337 patients. All the five biological agents proved to be significantly superior to placebo. Infliximab showed the highest odds ratio (OR) of 7.2 (95 % CI 3.68–13.19) compared to placebo, followed by infliximab-biosimilar with OR 6.25 (95 % CI 2.55–13.14), both assessed at week 24. No significant difference was found between infliximab-biosimilar and other biological treatments regarding their efficacy and safety.

**Conclusions:**

This is the first study which includes a biosimilar drug in the meta-analysis of biological treatments in AS. The results have proven the similar efficacy and safety profile of infliximab-biosimilar treatment compared to other biologicals.

## Introduction

So far adalimumab, etanercept, golimumab and infliximab have been approved by the European Medicine Agency (EMA) for the treatment of adults with severe, active ankylosing spondylitis (AS) who have responded inadequately to conventional therapy (see detailed description of the disease in Péntek et al. [1] in this Supplement).

In September 2013, the first biosimilar therapy, namely infliximab-biosimilar (CT-P13, trade names: Remsima and Inflectra) was licensed in the EU for the treatment of AS. The results of a Phase 1, multicenter, double-blind randomized controlled trial (RCT) with infliximab-biosimilar (called the PLANETAS study) were published in May, 2013 [2]. The trial was designed to demonstrate pharmacokinetic equivalence and efficacy and safety comparability of infliximab-biosimilar (CT-P13) and the originator infliximab in active AS patients. The RCT was conducted at 46 sites across 10 countries in Europe, Asia and Latin America between November, 2010 and December, 2011. Altogether, 250 patients were enrolled in the study. Besides pharmacokinetics, the proportions of patients achieving 20 and 40 % improvement according to the assessment of Spondyloarthritis International Society^1^ response criteria (ASAS20 and ASAS40) at weeks 14 and 30 were the endpoints to assess efficacy [3]. (See the definition of ASAS response criteria in the Methods Section). No significant differences were found in the efficacy and safety of the originator infliximab and infliximab-biosimilar. According to the study results, ASAS20 and ASAS40 responses at week 30 were 70.5 and 51.8 % for infliximab-biosimilar and 72.4 and 47.4 % for originator infliximab, respectively. The authors concluded that pharmacokinetic, efficacy and safety profiles of the infliximab-biosimilar and the originator infliximab were equivalent in patients with active AS [2].

According to our knowledge, no meta-analyses have been published yet in AS, which compare the efficacy and safety of the infliximab-biosimilar treatment to the other biological drugs indicated in AS. Thus, the aim of this study was to carry out a systematic literature review and meta-analysis of published RCTs in order to compare the efficacy and safety of infliximab-biosimilar to adalimumab, etanercept, golimumab and infliximab in AS.^2^

Besides the PLANETAS trial, no other RCTs, presenting head-to-head comparison of biologicals, have been published yet in this diagnosis [4]. Due to the difference in comparators across the trials (infliximab-biosimilar is compared to infliximab in the PLANETAS study, while other biologicals are compared to placebo), traditional methods cannot be applied for the comparison. Therefore, we used an indirect comparison method, namely mixed treatment comparison (MTC) to evaluate the efficacy and safety of biological treatments. MTC permits indirect comparisons between study drugs with different comparators as well [5, 6].

## Methods

### Treatments

In the current analysis adalimumab, etanercept, golimumab and infliximab are considered as comparators of infliximab-biosimilar as these biologicals are recommended by the EMA for the treatment of AS. Only doses recommended by the EMA were considered in the analysis: adalimumab (40 mg every other week as a subcutaneous injection); etanercept (25 mg twice weekly, or 50 mg once weekly as a subcutaneous injection); golimumab (50 mg once a month as a subcutaneous injection); infliximab (5 mg/kg at 0, 2, 6 weeks and then every 6–8 weeks as intravenous infusions over a 2-h period) and infliximab-biosimilar (CT-P13) (5 mg/kg at 0, 2, 6 weeks and then every 6–8 weeks as intravenous infusions over a 2-h period).

### Literature search

Electronic databases (Medline and Cochrane Library) as well as references of retrieved articles were searched. The Cochrane highly sensitive search strategy [7] was applied to identify randomized controlled publications and was combined with the disease (ankylosing spondylitis, ankylosing spondyloarthritis, spondyloarthritide) and drug names for adalimumab, etanercept, golimumab and infliximab.^3^ We carried out the search for the period between November 1, 2005 and August 20, 2013. To identify RCTs from earlier years, we relied on the systematic review of McLeod et al. [8] published in 2007, which assessed the comparative clinical effectiveness of adalimumab, etanercept and infliximab for the treatment of AS. A separate search was carried out to identify RCTs with the biosimilar agent, using its generic name (CT-P13) as search term, and in this case no further restrictions were applied.

### Exclusion and inclusion criteria

Double-blind RCTs in AS with parallel design, with full paper obtainable were included. Non randomized or uncontrolled studies, observational studies, case series, letters to editor, studies with no abstracts or with conference abstracts only were not included. A further inclusion criterion was that AS patients, diagnosed based on the modified New York criteria [9], in at least one arm of the trial must receive adalimumab, etanercept, golimumab, infliximab or infliximab-biosimilar treatment at the labelled dose. Studies which examined only off-label doses, or other than the suggested administration (e.g. infliximab combined with methotrexate) studies reporting solely on laboratory measures aimed at investigating disease, or treatment mechanisms and which do not report relevant clinical outcomes were excluded. Studies involving patients younger than 18 years were also excluded, as well as pilot studies.

### Data extraction

We used the same data extraction process and quality assessment of the RCTs as in our previous study in which we assessed the efficacy and safety of infliximab-biosimilar in another inflammatory rheumatic disease, rheumatoid arthritis (RA). Details have been published elsewhere [10]. In brief, data on study design, patients’ demographic and morbidity characteristics, treatment interventions, endpoints and duration of follow-up were subtracted. The quality of selected studies was evaluated using the JADAD-score [11].

### Endpoints

The proportions of patients with ASAS20 response at weeks 12 and 24 were used as efficacy endpoints in the meta-analysis of AS trials. The ASAS20 improvement criteria requires improvement of ≥20 % and ≥1 unit in at least 3 of 4 well-defined specific domains (patient global assessment, pain, function and inflammation) on a scale of 10 and no worsening of ≥20 % and ≥1 in remaining domain on a scale of 10 [3]. To evaluate the safety of biological therapies, the occurrence of serious adverse events at week 24 was used as a safety endpoint in the analysis. We could not carry out the safety analysis at week 12, as the infliximab-biosimilar study presented safety results only at week 30 [2].

### Meta-analysis

Mixed treatment comparison (MTC) was applied in the analysis [5, 6]. We estimated the posterior densities for all unknown parameters using MCMC (Markov chain Monte Carlo) for each model in WinBUGS version 1.4.3 (MRC Biostatistics Unit, Cambridge, UK). We applied a random effects model to estimate the odds ratios (OR) as the measure of relative treatment effect. We also present the 95 % credibility intervals (CI) which contain the true value of OR with 95 % probability.

## Results

### Literature review

Our literature search for the period between November 1, 2005 and August 20, 2013 yielded 336 potential citations for RCTs. Among them seven RCTs in AS with the target drugs of our study were identified. Five of them met our inclusion criteria [12–16]. One study was not enrolled as it examined off-label infliximab therapy (3 mg/kg) [17]. To have comparable results, one study was excluded as infliximab was given in combination with methotrexate [18]. Till November 2005, nine RCTs identified by the systematic review of McLeod et al. [8] were screened for eligibility. Seven of them met our enrollment criteria, and were included in the current meta-analysis [19–25]. (One study [26] was excluded as it examined the effect of etanercept at week 6, and another study was published later in a scientific journal by van der Heijde et al. [13], which was identified by our search as well in the Medline database). The search for infliximab-biosimilar did not identify any other RCT than the PLANETAS trial [2].

Thus, altogether 13 studies were included in the meta-analysis. Eight of them were 12-week trials: one with infliximab [24], five with etanercept [13–15, 21, 22] and two with adalimumab [12, 20]. Five of 13 studies were at least 24-week trials: one with infliximab [25], one with adalimumab [19], one with etanercept [23], one with golimumab [16] and one with infliximab-biosimilar [2].

The main characteristics of the RCTs, i.e. the number of patients enrolled, the treatment arms and the JADAD-scores are presented in Table 1.

**Table 1:** Characteristics of included studies

Studies	N	Week	Treatment	Mean age, years	Mean disease duration, years	Baseline BASDAI score^b^ (0–10)	JADAD-score
Park et al. [2] PLANETAS	250	30	(1) Infliximab-biosimilar 5 mg/kg at week 0, 2, 6, 14, 22 *n* = 125	38.0^a^	NR	6.8^a^	5
			(2) Infliximab 5 mg/kg at week 0, 2, 6, 14, 22 *n* = 125	38.0^a^		6.6^a^	
Braun et al. [24]	70	12	(1) Infliximab 5 mg/kg at week 0, 2, 6 *n* = 34	40.6	16.4	6.5	5
			(2) Placebo *n* = 35	39.0	14.9	6.3	
Van der Heijde et al. [25] ASSERT	279	24	(1) Infliximab 5 mg/kg at week 0, 2, 6, 12, 18 *n* = 201	40.0^a^	7.7^a^	6.6^a^	5
			(2) Placebo *n* = 78	41.0^a^	13.2^a^	6.5^a^	
Huang et al. [12]	344	12	(1) Adalimumab 40 mg eow *n* = 229	30.1	8.1	6.0	5
			(2) Placebo *n* = 115	29.6	7.7	6.2	
Van der Heijde et al. [19] ATLAS	315	24	(1) Adalimumab 40 mg eow *n* = 208	41.7	11.3	6.3	5
			(2) Placebo *n* = 107	43.4	10.0	6.3	
Maksymovich et al. [20]	82	12	(1) Adalimumab 40 mg eow *n* = 38	41.9	14.5	6.2	4
			(2) Placebo *n* = 44	40.0	12.1	6.5	
Gorman et al. [21]	40	16 (4 months)	(1) Etanercept 25 mg twice weekly *n* = 20	38.0^a^	15^a^	NR	5
			(2) Placebo *n* = 20	39.0^a^	12^a^		
Calin et al. [22]	84	12	(1) Etanercept 25 mg twice weekly *n* = 45	45.3	15.0	61.0^c^	5
			(2) Placebo *n* = 39	40.7	9.7	58.6^c^	
Davis et al. [23]	277	24	(1) Etanercept 25 mg twice weekly *n* = 138	42.1	10.1	58.1^c^	5
			(2) Placebo *n* = 139	41.9	10.5	59.6^c^	
van der Heijde et al. [13]	356	12	(1) Etanercept 50 mg once weekly *n* = 155	41.5	9.0	62.4^c^	4
			(2) Etanercept 25 mg twice weekly *n* = 150	39.8	10.0	59.4^c^	
			(3) Placebo *n* = 51	40.1	8.5	61.1^c^	
Barkham et al. [14]	40	12	(1) Etanercept 25 mg twice weekly *n* = 20	40.8	11^a^	6.1	4
			(2) Placebo *n* = 20	39.4	20^a^	5.5	
Dougados et al. [15] SPINE	82	12	(1) Etanercept 50 mg once weekly *n* = 39	46.0	19	64.0	5
			(2) Placebo *n* = 43	48.0	23	58.0	
Inman et al. [16]	356	24	(1) Golimumab 50 mg every 4 weeks *n* = 138	38.0^a^	11.0^a^	6.6^a^	5
			(2) Golimumab 100 mg every 4 weeks *n* = 140	38.0^a^	9.5^a^	7.0^a^	
			(3) Placebo *n* = 78	41.0^a^	16.0^a^	6.6^a^	

*NR* not reported, *eow* every other week

^a^Median

^b^Bath Ankylosing Spondylitis Disease Activity Index [32]

^c^Scale: 0–100

### Mixed treatment comparison meta-analysis: efficacy and safety

#### Efficacy

The infliximab-biosimilar study and Inman et al. [16] golimumab study presented ASAS20 results at week 14, and the Gorman et al. [21] etanercept study at week 16 (4 months). These studies were pooled with trials presenting results for week 12. In this way, results of twelve studies involving 2,395 patients were analyzed for ASAS20 endpoint at week 12. All biologicals were found to be significantly superior to placebo. Compared to placebo, infliximab showed the highest OR for ASAS20 response at week 12, OR 6.74 (3.81–11.3), followed by infliximab-biosimilar OR 6.39 (2.75–12.78) and golimumab OR 5.7 (2.88–10.44). Results are presented in Table 2.

**Table 2:** The efficacy of infliximab-biosimilar and other biologicals compared to placebo in AS, results of the mixed treatment comparison

Substance	ASAS20 at week 12, OR (95 % CI)	ASAS20 at week 24, OR (95 % CI)	Serious adverse events OR (95 % CI)
Adalimumab	4.65 (3.29–6.43)	4.81 (2.67–8.18)	1.57 (0.27–5.72)
Etanercept	4.35 (3.09–5.96)	4.76 (2.73–7.81)	2.36 (0.64–6.58)
Golimumab	5.7 (2.88–10.44)	4.53 (2.32–8.22)	0.69 (0.14–2.1)
Infliximab	6.74 (3.81–11.3)	7.2 (3.68–13.19)	2.71 (0.35–12.03)
Infliximab-biosimilar^a^	6.39 (2.75–12.78)	6.25 (2.55–13.14)	2.31 (0.17–11.43)

^a^Results for weeks 14 and 30 were available and considered for infliximab-biosimilar

Four studies reported ASAS20 response at week 24. The infliximab-biosimilar RCT presented ASAS20 results at week 30. However, patients in this trial received the same number of infusions as patients in the 24-week infliximab study. Therefore, we pooled these five studies involving 1,337 patients in the analysis of ASAS20 response at week 24.

At week 24, infliximab showed the highest ORs compared to placebo [OR 7.2 (95 % CI 3.68–13.19)], followed by infliximab-biosimilar [OR 6.25 [95 % CI 2.55–13.14)] and adalimumab [OR 4.81 (95 % CI 2.67–8.18)], see Table 2. All biologicals were found to be significantly superior to placebo.

**Fig. 1:**
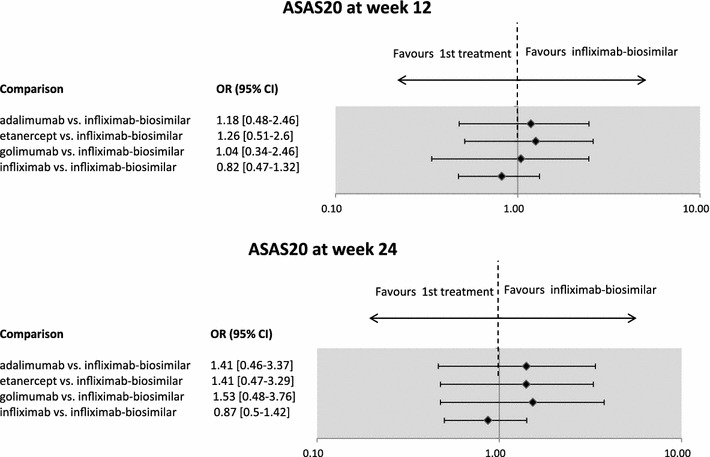
Efficacy of infliximab-biosimilar compared to other biologicals in AS, results of mixed treatment comparison (ASAS20 response at weeks 12 and 24). Results for weeks 14 and 30 were available and considered for infliximab-biosimilar. Note: the Figure presents odds ratios (OR) between treatments. If the point estimate is greater than 1, then the biosimilar treatment is more effective (although not necessarily statistically significantly more effective) compared to the originator biologicals. Credibility intervals provide information on whether the difference between treatments is statistically significant. If the CI contains the value 1, the difference is not statistically significant

The results of the pairwise comparison did not show significant differences between the efficacy of infliximab-biosimilar and the other biologicals in terms of ASAS20 response: neither at week 12, nor at week 24 (see Fig. 1).

#### Safety

The occurrence of severe adverse events (AE) was examined at week 24. Five AS studies involving 1,337 patients reported the occurrence of severe AEs at week 24. At this endpoint the lower ORs are in favor of biologicals, as the lower the OR, the lower the chance of the occurrence of serious AEs compared to placebo.

Golimumab gave the lowest ORs compared to placebo [OR 0.69 (95 % CI  0.14–2.1)], followed by adalimumab [OR 1.57 (95 % CI 0.27–5.72)] and infliximab-biosimilar [OR 2.31 (95 % CI 0.17–11.43)]. We have not found significant difference between placebo and biological treatments regarding safety.

**Fig. 2:**
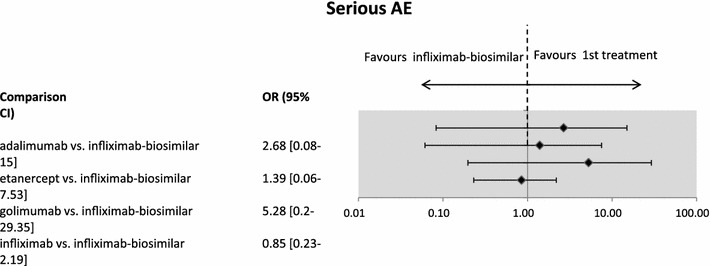
The safety of infliximab-biosimilar compared to other biologicals in AS: serious adverse events (AE). Results for week 30 were available and considered for infliximab-biosimilar. Note: the Figure presents odds ratios (OR) between treatments. If the point estimate is lower than 1 then the biosimilar treatment is safer (although not necessarily statistically significantly safer). Credibility intervals provide information on whether the difference between treatments is statistically significant. If the CI contains the value 1, the difference is not statistically significant

Regarding the pairwise comparison of the treatments, we found no significant difference in the safety of infliximab-biosimilar and other biological treatments (see Fig. 2).

## Discussion

Our study, based on the meta-analysis of available RCTs, involving 2,395 AS patients at week 12 and 1,337 AS patients at week 24, has demonstrated that there is no significant difference in the efficacy of infliximab-biosimilar and other biological drugs in terms of ASAS20 improvement. The results showed no significant differences in the safety of infliximab-biosimilar and biologicals either.

Some of the former meta-analyses synthetized the evidence of a single biological agent against placebo [27–29]. All these studies concluded that biological agents were superior to placebo. Thaler et al. [30] in their extensive review (2012) compared the efficacy and safety of 12 biologicals in seven inflammatory diseases, including AS, based on literature published between January 2009 and October 2011. However, they have not presented results regarding the indirect comparison of available treatments in AS.

McLeod et al. [8] assessed the comparative clinical effectiveness and cost-effectiveness of adalimumab, etanercept and infliximab for the treatment of AS. The authors carried out traditional direct and indirect comparisons of the treatments. Nine placebo-controlled RCTs were included in their meta-analysis. According to their findings the difference between biologicals was not significant.

Mixed treatment comparison (MTC) was used by Migliore et al. [4] and Shu et al. [31].

Shu et al. compared the effectiveness of different doses of adalimumab, golimumab and infliximab in terms of ASAS20 response at week 12. Fourteen RCTs were included in their analysis.^4^ All drug dosages applied in the RCTs were assessed, while we focused only on treatment arms with the doses recommended by the EMA. Nevertheless, authors came to the same conclusion as us, namely that infliximab 5 mg/kg at 0, 2, 6 weeks was the best efficacious therapy [OR 6.53 (95 % CI 3.35, 11.61)] compared to placebo [31]. No significant differences were found between the biological treatments either.

Migliore et al. [4] compared ASAS20 response at week 24 between biological agents. Three RCTs were included in their analysis, as the 24-week golimumab RCT and the recently published RCT with infliximab-biosimilar were not included [4]. The authors found no significant differences when comparing directly one biological agent against another. When compared with placebo, infliximab increased the probability of response by 7 times (OR 6.8), adalimumab by 4 times (OR 4.4), and etanercept by 5 times (OR  4.9). These results are in line with our findings, and confirm the validity of our study.

We have to acknowledge some limitations of our study. First, a potential weakness of this meta-analysis arises from the fact that the trials from which data are combined are likely to differ in their design. For example, the infliximab-biosimilar study reports efficacy and safety results at week 14 and 30 while most of the others do so for week 12 and 24; that is infliximab-biosimilar results are from 2 to 6 weeks later, respectively. However, we do not expect strong bias related to this difference as patients in the infliximab-biosimilar study received the same number of infusions as patients in the infliximab study. Also, patient characteristics (age, disease duration, baseline BASDAI score) varied slightly across studies. Furthermore, only the primary efficacy outcome was assessed in this analysis (ASAS20). Other efficacy endpoints were not investigated as, on the one hand, some of the RCTs have not reported ASAS40, and on the other hand, the infliximab-biosimilar RCT did not assess another activity score, the 50 % improvement of the initial disease activity score of the Bath ankylosing spondylitis disease activity index (BASDAI50). Also, the safety analysis was carried out only for the occurrence of serious adverse events at week 24, since the infliximab-biosimilar study presented safety results only at week 30. In this way only five RCTs were included in the safety analysis. Despite these limitations, we believe that our analysis contributes with important results to the evidence-based health care evaluation of AS that might support clinical as well as financial decision making.

In conclusion, infliximab-biosimilar has recently been approved by the European Medicines Agency for the treatment of adults with active AS and this first meta-analysis suggests that it is similar in both efficacy and safety to other biologicals. Further head-to-head comparisons, continuous data collection and benefit-risk assessment might confirm our results.

## Footnotes

1 Former Asessment in Ankylosing Spondylitis.

2 The search dates were November 1, 2009 to August 20, 2013. Certolizumab pegol was registered for the treatment of AS on 19 September 2013, thus it was not included in our analysis.

3 (“Ankylosing spondylitis” OR “ankylosing spondyloarthritis” OR “spondyloarthritide”) AND (“adalimumab” OR “infliximab" OR “golimumab" OR “etanercept") AND [(randomized controlled trial[pt] OR controlled clinical trial[pt] OR randomized[tiab] OR placebo[tiab] OR “clinical trials as topic”[MeSH Terms] OR randomly[tiab] OR trial[ti]) NOT (“animals”[MeSH Terms] NOT “humans”[MeSH Terms])] AND (“2005/11/01”[PDAT]: “2013/08/20”[PDAT]).

4 Shu et al.’s study included two additional RCTs, which were not included in our analysis—for further explanation, see the Results Section.
